# Lymphocytes as Liver Damage Mirror of HCV Related Adipogenesis Deregulation

**DOI:** 10.1371/journal.pone.0092343

**Published:** 2014-03-21

**Authors:** Antonella Minutolo, Beatrice Conti, Sandro Grelli, Carmela Viscomi, Giancarlo Labbadia, Clara Balsano

**Affiliations:** 1 Laboratory of Molecular Virology and Oncology, Francesco Balsano Foundation, ex A. Cesalpino Foundation, Rome, Italy; 2 Institute of Biology and Molecular Pathology (IBPM) – CNR (National Research Council), Rome, Italy; 3 U.O.C. Clinical Microbiology, Tor Vergata Hospital, Rome, Italy; 4 Department of Clinical and Medical Therapy, “Sapienza” University of Rome - Umberto I Hospital, Rome, Italy; Istituto Nazionale per le Malattie Infettive, Italy

## Abstract

Hepatitis C virus infection leads to a wide spectrum of liver diseases ranging from mild chronic hepatitis to end-stage cirrhosis and hepatocellular carcinoma. An intriguing aspect of the HCV infection is its close connection with lipid metabolism playing an important role in the HCV life cycle and in its pathogenesis. HCV is known to be a hepatotropic virus; however, it can also infect peripheral blood mononuclear cells (PBMCs). The goal of the current investigation is to compare the adipogenesis profile of liver tissues to lymphocytes of HCV infected patients, in order to understand if PBMCs may reflect the alterations of intracellular pathways occurring during HCV-related liver steatosis. Using the Human Adipogenesis PCR Array, gene expression was analyzed in liver samples and PBMCs of chronic HCV+, HBV+ and Healthy Donors (HDs) patients. We observed a similar modulation of lipid metabolism in HCV+ and HBV+liver tissues and lymphoid, cells suggesting that PBMCs reflect the liver adipogenesis deregulation related to infection, even if the two viruses have a different impact in the regulation of the adipogenesis mechanisms. In particular, some genes involved in lipid metabolism and inflammation, as well as in cell transformation, were up-regulated, in a similar way, in both HCV models analyzed. Interestingly, these genes were positively correlated to virological and hepatic functional parameters of HCV+ patients. On the contrary, HBV+ patients displayed a completely different profile. PBMCs of HCV+ patients seem to be useful model to study how HCV-related lipid metabolism deregulation occurs in liver. The obtained data suggest some molecules as new possible biomarkers of HCV-related liver damage progression.

## Introduction

The Hepatitis C virus (HCV) infection affects, approximately, 170 million individuals, about 3% of the world's population [1]. The severity grade and the speed of liver damage progression are defined by several cofactors such as age, gender, the host's genetic background, alcohol and drugs consumption, immune status, co-infections and the metabolic status [2], [3]. HCV infects both hepatocytes and peripheral blood mononuclear cells (PBMCs) [4], [5], [6].

The HCV infection is characterized by specific clinical abnormalities of lipid metabolism, such as hypo-beta-lipoproteinaemia and liver steatosis [7]. The presence of steatosis and the progression throughout fibrosis are some of the main histological features of HCV infected patients [8], [9]. The viral deregulation of lipid metabolism affects liver disease progression and the response to antiviral therapies [10]. The density of circulating HCV particles in the blood of chronically infected patients is very heterogeneous. The very low density of some particles has been attributed to an association of the virus with apolipoprotein-beta (apoB) positive and triglyceride rich lipoproteins (TRL) likely, resulting in hybrid lipoproteins, known as lipo-viro-particles (LVP). LVPs contain the viral envelope glycoproteins E1 and E2, capsid and viral HCV-RNA. The specific infectivity of these particles has been shown to be higher than the infectivity of particles of higher density [11], [12]. Moreover, lipids play an important role in HCV life cycle: the viral entry is partly mediated by lipoprotein receptors (LDLR) [13]; the maturation and secretion of viral particles are mediated by the Apolipoprotein A–I [14] and the Very Low Density Lipoprotein (VLDL).

Currently, it is clear that HCV affects more than one aspect of the lipoprotein metabolism and the associated liver damage. The HCV-related lipid deregulation causes overexpression of different hepatic enzymes: hormone sensitive Lipase (LIPE), lipoprotein lipase (LPL) and Leptin [15]. Accordingly, patients exhibit serum lipid abnormalities, such as low levels of: LDL [16], [17], Resistin (RETN) [18], Adiponectin (ADIPOQ) [19] and Adipogenin (ADIG) [20]. The genes involved in the lipid *de novo* synthesis and beta oxidation, such as the nuclear receptor Liver X Receptor alpha (LXR alpha), the Sterol regulatory element binding transcription factor 1 (SREBP1c), the Fatty acid synthase (FASN) and the Acetyl CoA carboxylase Beta (ACACB) respectively, are associated with HCV infection, too [21], as well as Fatty acid binding protein 4 (FABP4) that modulates inflammatory and metabolic response that is mainly expressed in adipocytes and macrophages [22].

We asked if PBMCs might be the mirror of the adipogenic deregulation occurring in the liver tissue, during HCV infection. Thus, we compared the adipogenesis profile of liver tissues to lymphocytes of HCV infected patients.

## Experimental Procedures

### Patients recruitment and biological samples collection

This research is born as a descriptive and observational study aimed to characterize adipogenesis profile in HCV+, HBV+ patients and healthy donors. The study protocol was approved by the Ethics Committee of Sapienza University, and written informed consent was obtained from each subject at the time of enrollment. “(Rif. 2534/26.07.2012 n. prot.717/12)”. 40 HCV, 20 HBV-infected patients and 20 healthy donors (HDs) were enrolled in an open study by the Department of Public Health and Infectious Disease, University of Rome “La Sapienza” and UOC Clinical Microbiology, “Tor Vergata” Hospital of Rome. Exclusion criteria for HCV and HBV patients were: 1) previous treatment with antiviral therapy, immunosuppressive drugs, and/or regular use of drugs influencing lipid metabolism and/or oxidative stress; 2) advanced cirrhosis; 3) hepatocellular carcinoma; 4) other causes of liver disease or mixed etiologies; 5) human immunodeficiency virus infection; 6) active intravenous drug addiction, 7) alcohol consumption. The current study was performed in accordance with the principles of Good Clinical Practice, the principles of the Declaration of Helsinki, and its appendices, and local and national laws. Liver and Blood samples were collected at the same time and blood samples were used to isolate PBMCs through a density gradient according to the standard Ficoll-Hypaque (Pharmacia) technique. Separate scores for steatosis, lobular inflammation, and hepatocellular ballooning and to stage fibrosis from 0 to 4 [23]. Biopsies of HCV patients were classified for grade and stage according to the Scheuer system [24].

### Viral Load and Qualitative detection of antibodies to the Hepatitis C virus

HCV RNA viral load (VL) levels were determined using a quantitative ultrasensitive reverse transcription-polymerase chain reaction assay (Roche Diagnostics).

To detect anti-HCV antibodies in human sera (recombinant proteins NS3-NS4 and anti-capsid antibody) was used The Access (Beckman Coulter) HCV Ab PLUS assay immunoenzymatic method (Biorad).

### HBV viral load, HBeAg and HBsAg detection

Qualitative HBeAg and Quantitative HBsAg levels were determined by using the Roche Cobas reagent kits (Roche Diagnostics). The HBV DNA viral load (IU/mL) was determined by using Cobas AmpliPrep/Cobas TaqMan HBV Test (Roche Diagnostics).

### Cell culture, Constructs and Reagents

The human hepatoma-derived cell line Huh7.5 were grown in Dulbecco's modified Eagle's medium (DMEM) supplemented with 10% fetal bovine serum, 100 U/ml penicillin and 100 mg/ml streptomycin and 2 mM L-glutamine (Lonza).

J6/JFH1 cDNA (kindly provided by C. Rice, Rockefeller University) was used to generate the HCV cell model. Antibodies anti human KLF15, KLF3, TCF7L2 (Abcam), Adipsin, Adipogenin, Twist 1, beta Actin (Santa Cruz Biotechnology, Santa Cruz, CA), HCV NS5a (Austral Biologicals) were used for immunofluorescence assay. Detection was achieved using horseradish peroxidase–conjugate secondary antibody (Jackson ImmunoResearch Laboratories, West Grove, PA). ECL Plus Western and ECL-Hyperfilm (GE Healthcare Life Sciences) were used. Specific neutral lipids were stained with BODIPY (Invitrogen).

### Replication and infection assays

The chimeric J6/JFH1 virus was generated and used according to Lindenbach BD and collegues [25]. Huh7.5 cells were infected (MOI = 0.1) 1 day after seeding. After 3 days cells were harvested and processed for RNA and protein extractions.

The lipid accumulation was evaluated through the fluorescent probe BODIPY used as a stain for neutral and non-polar lipids in immunofluorescent assay.

### Immunofluorescence

Cells were grown on coverslips and fixed with 4% paraformaldehyde followed by permeabilization with 0.1% Triton X-100 in phosphate-buffered saline (PBS). Primary antibodies were incubated for 1 h at room temperature and visualized by conjugated secondary antibodies (invitrogen). Coverslips were mounted in SlowFade-Anti-Fade (Invitrogen) and examined under a confocal microscope (Leica TCS SP2)

### Samples for mRNA expression analysis

Frozen liver biopsies tissues RNA were prepared with Trizol reagent (Invitrogen). Complementary DNA synthesis was generated from 2 μg of total RNA using the reverse transcription kit (Promega).

### Human Adipogenesis PCR Array

To investigate a wide spectrum of genes involved in lipid metabolism the Human Adipogenesis RT^2^ Profiler PCR Array (Qiagen) was used. 84 “metabolic” genes were analyzed in addition to positive control genes (beta actin and Ribosomal protein). Real-time PCR was performed with 7500 Fast Real-Time (Applied Biosystems) using Sybr green detection. Results were analyzed by RT^2^ Profiler PCR Array Data Analysis. Transcriptional levels of genes were shown as fold change *vs* Healthy Donors, values >1.50 or <0.5 were considered significantly modified. All dataset was submitted in Geo Repository http://www.ncbi.nlm.nih.gov/geo. Accession number: GSE54432

### Western blot analysis

For protein extraction, liver biopsies were homogenized and maintained on ice. Cells were collected and lysed and the proteins extracted were analyzed through SDS–PAGE and probed with different antibodies followed by detection with ECL plus (GE Healthcare).

### Statistical Analysis

Statistical analysis of data was performed using the SPSS statistical software system (17.0 Windows). The independent samples t-test analysis was performed; for non-parametric correlation, Spearman's rho correlation coefficient was calculated by Bonferroni's correction.

## Results

### Patients' characteristics

For this pilot study, 40 naïve chronic HCV+ (72% genotype 1; 28% genotype 2), 20 HBV+ patients and 20 Healthy Donors (HDs) matched by age and gender, have been consecutively enrolled. Neither infected patients nor HDs were under any treatment at the moment of the enrollment. **Table 1** **,** **2** **and** **3** highlight the clinical features of all patients studied. 27.3 % of HCV+ patients had a F2/F3 liver fibrosis, the remaining had F0-F1 liver fibrosis. Regarding the liver state of HBV+ patients, more than 80% had a F0/F1 grade of fibrosis (81,7%), the remaining had F2 status. Moreover 55% HBV population resulted HBeAg positive and 45% HBeAg negative. No steatosis was ecographically detectable in HD patients.

**Table 1:** Biochemical data of HCV+ and Healthy Donors (HDs).

	HCV (40)	HDs (20)	*P^1^*
Sex (F/M)	25/15	9/11	
Age (years)	53.7±15.3	48.3±6.0	NS
Body weight (kg)	64.6±11.6	64.7±5	NS
BMI (kg/m2) (≤25)	24.6±4	24.7±0.6	NS
HOMA-IR (<2)	2.6±2.0	1.0±1.2	<0.05
cholesterol (81.1–235.5 mg/dL)	172.3±9.7	185.7±26.2	NS
HDL (34.7–56 mg/dL)	62.4±22.6	56.7±4.2	NS
LDL (54.8–120 mg/dL)	89.9±33.8	101.3±13.4	NS
Triglycerides (45.1–235 mg/dL)	91.7±12.9	90.7±15	NS
Ferritin (30–400 μg/L)	209.6±17	201.0±15.5	NS
AST (9–45 IU/mL)	50.6±14.1	29.7±10.2	<0.05
ALT (10–40 IU/mL)	71.6±9.7	26.3±2.3	<0.05
GGT (8–61 IU/mL)	55.1±4.2	50.1±1.0	NS
AP (40–129 IU/mL)	86.7±8.4	65.3±13.6	<0.05
Total bilirubin (<1.20 mg/dL)	0.7±0.3	0.55±0.1	NS
hsCRP (0.1–0.6 g/L)	1.8±0.3	0.4±0.2	<0.05
VES (0–25 mm/h)	13.8±1.5	10.0±0.5	NS
Glucose (73.9–109.9 mg/dL)	102.6±53.1	88.3±10.1	<0.05
Insulin (2.6–24.90 μU/mL)	9.3±3.8	4±1.6	<0.05
Hyaluronic acid (ng/ml)	106.3±45.3	51,7±18.3	NS

Footnotes: *P^1^* t-test HCV *vs* HDs.

**Table 2:** Biochemical data of HBV+ patients.

	HBV pts (20)		*P^2^*
Sex (F/M)	12-ago		
Age (years)	51,5±6.35		NS
Body weight (kg)	63,5±10.56		NS
Body mass index (kg/m2) (≤25)	24,86±5		NS
HOMA-IR (<2)	1,71±0.36		NS
Total cholesterol (81.1–235.5 mg/dL)	187±0.63		NS
HDL cholesterol (34.7–56 mg/dL)	60±23.55		NS
LDL cholesterol (54.8–120 mg/dL)	115,5±23.6		NS
Triglycerides (45.1–235 mg/dL)	106,6±15.9		NS
Ferritin (30–400 μg/L)	210,45±13		NS
AST (9–45 IU/mL)	46±5.7	HBeAg −	NS
	49±3.7	HBeAg +	NS
ALT (10–40 IU/mL)	38±9.7	HBeAg −	NS
	78±5.63	HBeAg +	<0.05
GGT (8–61 IU/mL)	53.1±6		NS
AP (40–129 IU/mL)	78,8±7.3		<0.05
Total bilirubin (<1.20 mg/dL)	0,56±0.12		NS
hsCRP (0.1–0.6 g/L)	1,69±0.2		<0.05
VES (0–25 mm/h)	15±1.9		NS
Glucose (73.9–109.9 mg/dL)	90,2±12		NS
Insulin (2.6–24.90 μU/mL)	1,11±2.6		NS
Hyaluronic acid (ng/ml)	86,36		NS

Footnotes: *P^2^* t-test HBV *vs* HDs.

**Table 3:** Virological data of HCV+ and HBV+.

HCV pts	HCV-RNA PCR 10^6^ (UI/mL)	2,11±27	<0.05*	*P^3^*
	HCV genotype	1a: 42% (19)		
		1b: 30% (13)		
		2c: 17% (5)		
		2a/2c: 11% (3)		
HBV pts	Log10 IU/mL	HBV DNA	HBsAg	
	HBeAg −	4,6±0.36	1,642±0,15	
	HBeAg +	7,3±0.25	9,459±0,85	
		<0.05*	<0.05*	*P^4^*

Footnotes: P^3^ HCV *vs* HDsP^4^ Anti HBeAg + *vs* Anti HBeAg -.

### The adipogenesis of chronic HCV infected patients

**Figure 1:**
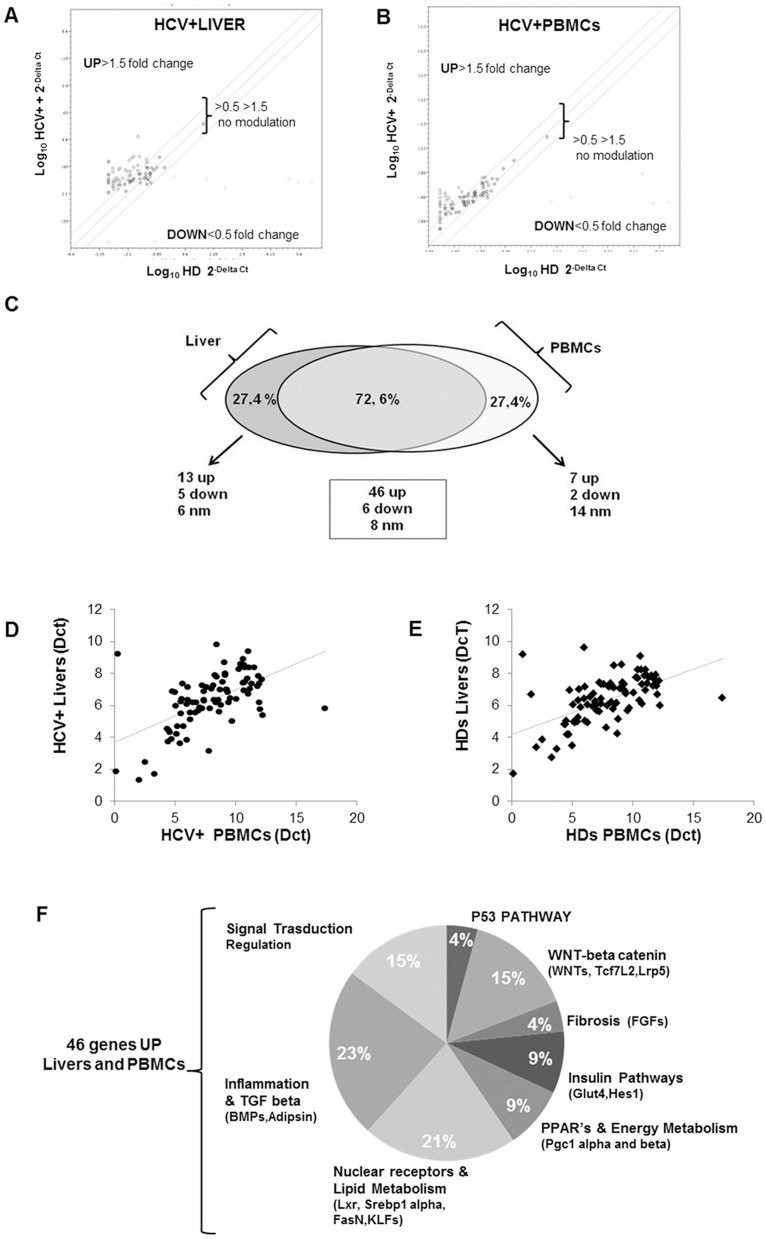
HCV affects lipid metabolism in livers and PBMCs of chronic HCV + patients. A) Dot Plot Array profile in HCV+ *vs* HDs liver biopsies and PBMCs (B). Transcriptional levels of genes are shown as fold change (HCV+ *vs* HDs). Values >1.50 or <0.5 were considered significantly modified. C) Percentage of the total adipogenesis gene transcripts modulated in chronic HCV+ patients (livers and PBMCs). D, E) Significant positive correlation between all genes in HCV+ livers and PBMCs (rho values 0.688 and 0.889 respectively, p<0.005). F) Percentage of functional genes groups UP modulated in HCV+ livers and PBMCs.

First of all, we performed the RT-PCR array to investigate the adipogenesis profile on HCV+ liver biopsies. The liver of HCV+ patients was characterized by a deep deregulation of genes involved in the control of glucose and lipid metabolism (**Figure 1A**). Interestingly, we obtained a similar gene expression profile in the HCV+ PBMCs (**Figure 1B**). It is intriguing to note the two experimental models have a similar behavior: the larger part of the analyzed genes was modulated in a similar way (**Figure 1C**). All the details of adipogenesis profile in chronic HCV+ PBMCs and liver tissues were shown in **Table S1**.

In particular, the analysis of the HCV+ biopsies and PBMCs compared to HDs profile, revealed that 72,6 % of the analyzed genes were modulated in the same manner: 46 (77%) genes were up-regulated, 6 (10%) down-regulated and 8 (13%) were unaffected by the virus. 27,4% of the studied genes were modulated in a different way (**Figure 1C** **and** **Table S1D**).

Spearman's rho statistical investigation showed a significant positive correlation (rho  = 0.688, p<0.001) among the genes analyzed, in both HCV+ liver biopsies and PBMCs (**Figure 1D**).

The same statistical evaluation was performed in HDs liver tissues and PBMCs, and gave us similar results (**Figure 1E**, rho = 0.889, p<0.001).

Moreover, here we want to underline that HCV *in vitro* model is characterized by a similar transcriptional modulation of some genes (Adig, Adipsin, KLF15, KLF3, TCF7L2, TWIST1) already analyzed in HCV+ liver tissues and PBMCs, supporting the belief that PBMCs might be a good substitute of liver tissues for the investigation of HCV-dependent metabolic derangement (see below).

At this point, we considered interesting to focus our attention on HCV+ up-regulated genes, because in the other two groups (down-regulated and no modulated genes) there weren't genes involved in the control of adipogenic pathways. Among the up regulated genes, 41% of them were transcriptional factors involved in glucose and lipid metabolism (9.5% insulin pathway, 9.5% energy metabolism and 22% nuclear receptors) and 59% in cell transformation (4% p53 pathways, 15% Wnt/beta catenin, 9% fibrosis, 8% signal transduction and regulation and 23% inflammation) (**Figure 1F**).

Moreover we have noticed that the 25% of modulated genes were significantly more expressed in the HCV + patients with elevated liver damage (F2/F3) in comparison to HCV+ F0/F1. (**Figure S1** and **Table S3**, p<0.05).

### The deregulation of adipogenesis is specifically due to HCV-infection

**Figure 2:**
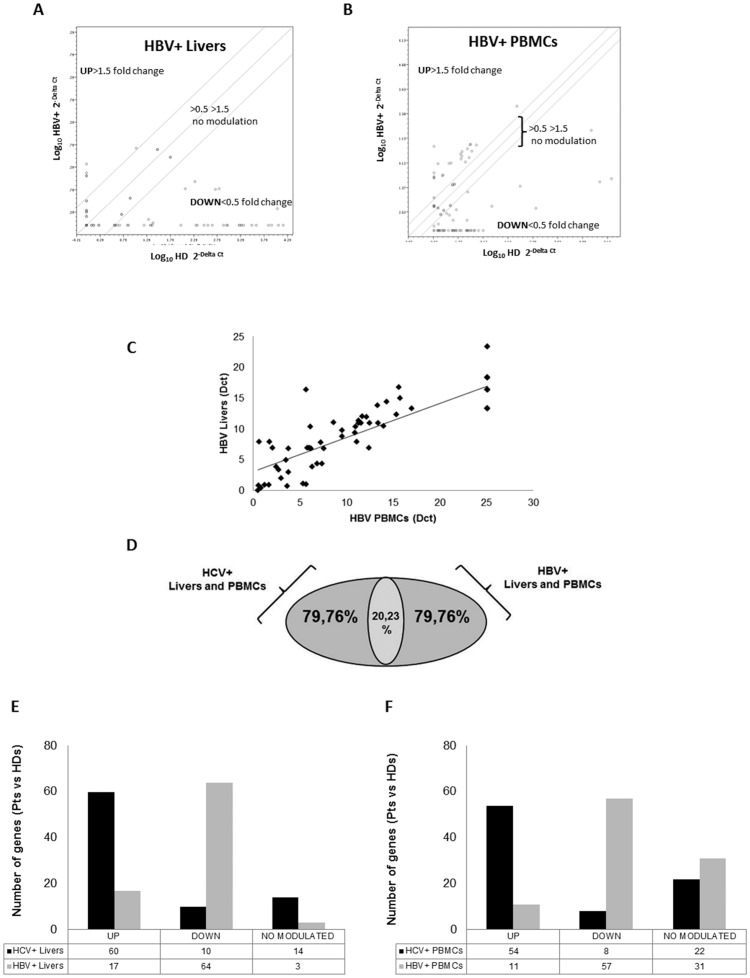
HCV and HBV infection adipogenesis profile. A, B) Dot Plot Array profile in HBV+ livers and PBMCs *vs* HDs. C) Significant positive correlation between all genes in HBV+ livers and PBMCs (rho values 0.759, p<0.05). D) Percentage of the total adipogenesis gene transcripts modulated in chronic HCV+ *vs* HBV+ patients' livers and PBMCs. E, F). Histogram plot comparing different number of genes modulation in HCV+ and HBV+ livers and PBMCs.

Since HCV infection causes a similar deregulation of adipogenesis in liver tissues and PBMCs, we wondered whether such alterations were specifically mediated by HCV. To this purpose, we performed the adipogenesis array in HBV+ Livers and PBMCS (**Figure 2A, B**). Also in this case we have appreciated a similar expression in livers and PBMCs (**Table S2**) and a direct significant correlation between gene expression in liver and lymphocytes. Spearman's rho statistical investigation showed a significant positive correlation (** rho  = 0.745) among the genes analyzed, in both HBV+ liver biopsies and PBMCs (**Figure 2C**). The HBV+ gene expression was greatly different from the profile displayed by HCV+.(**Figure 2D**) in fact 80% of genes showed a different expression in the two infection groups. (**Figure 2E, F**). Such data highlight the specificity of the adipogenesis profile of HCV infected patients.

### Possible novel biomarkers of HCV-dependent liver damage

**Figure 3:**
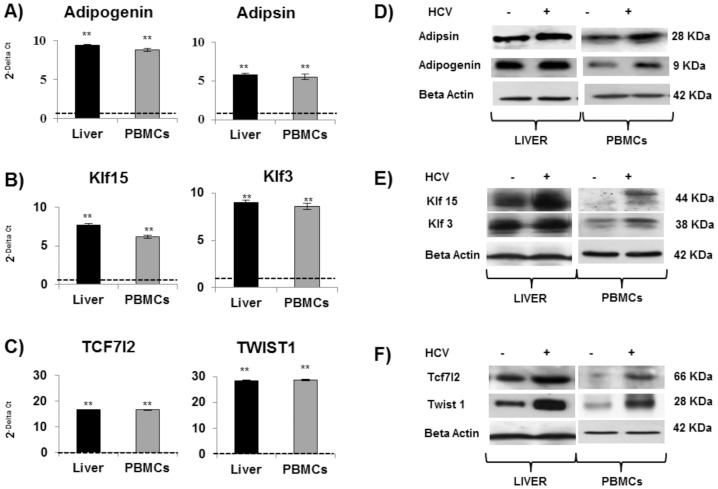
HCV up-regulates genes involved in lipid metabolism but with addictional role in inflammation, proliferation and transformation. Real-time analysis (mean values± S.D) of ADIG and Adipsin (A), KLF15 and KLF3 (B), TCF7L2 and TWIST 1 (C) are expressed as fold change, and normalized with an housekeeping gene. Asterisks indicate significant difference between HCV+ vs HDs (*p<0,05; **p<0.001) Adipogenin and Adipsin (D); KLF15 and KLF3 (E); TCF7L2 and TWIST 1 (F) protein levels in HCV+/HDs Liver and PBMCs. One experiment representative of all the independent experiments performed is shown. Beta-actin indicates the equal amount of protein loaded for each sample.

Some of the analyzed genes struck our attention because had never been directly associated with HCV infection. All the results, obtained the RT PCR-array approach, were confirmed by the relative mRNAs and protein expression levels (** p<0.001), (**Figure 3A and B**), and highlighted a strong up-regulation of two important transcription factors: Adipogenin (ADIG), Adipsin (CFD), Krüppel-like transcription factors 15 and 13 (KLF15 and KLF3) (**Figure 3C and D**) and TCF7L2 and Twist1 (**Figure 3E and F**).

### Deregulation of metabolic profile in *in vitro* system model of HCV infection

**Figure 4:**
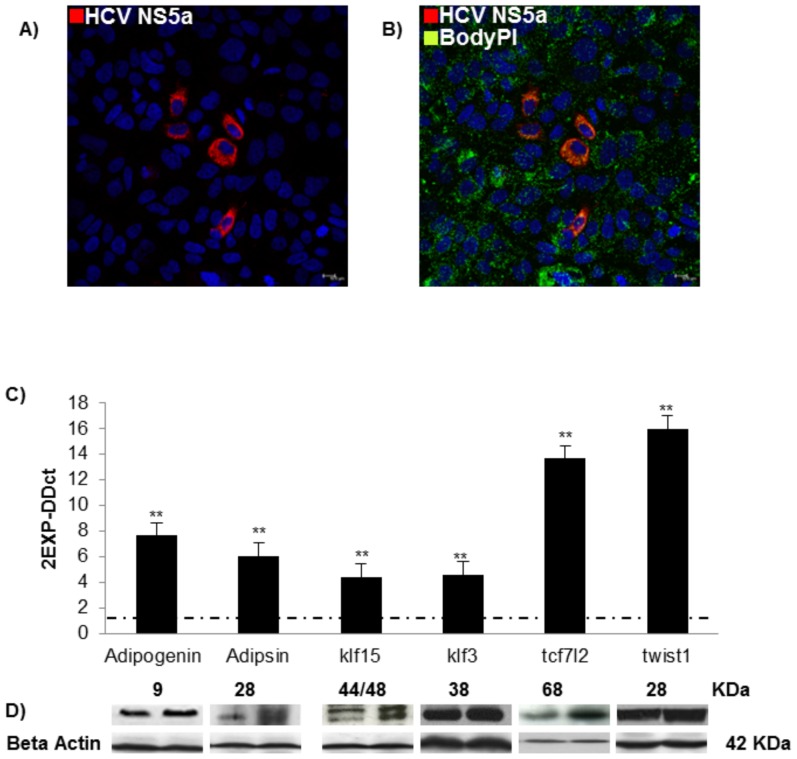
In *in vitro* HCV model up-regulates genes involved in lipid metabolism. Immunofluorescence assay HCV NS5A (red) (A) and lipid accumulation (green) (B). C) Real-time analysis (mean values± S.D) of ADIG, Adipsin, KLF15, KLF3, TCF7L2 and TWIST 1 are expressed as fold change and normalized with a housekeeping gene. Asterisks indicate significant difference between J6/JFH1 HuH7.5 vs HuH7.5 (*p<0,05; **p <0.001). D) protein levels in HCV+/HDs Adipogenin and Adipsin, KLF15, KLF3, TCF7L2, TWIST 1in HuH7.5 and J6/JFH1 HuH7.5. One experiment representative of 5 independent experiments performed is shown. Beta-actin indicates the amount of protein loaded for each sample.

To confirm that the expression of modulated genes in PBMCs reflected what happens in hepatocytes during HCV infection, we used an HCV *in vitro* model. 72 hrs post infection we revealed the presence of HCV-protein NS5A (**Figure 4A**) and a high intracellular fat accumulation in infected hepatocytes (**Figure 4B**).

The HCV *in vitro* model had a similar modulation of the previously analyzed genes (**Figure 4C**, ** p<0.001), supporting the idea that PBMCs may possibly be used as a substitute of liver tissues for metabolic investigation, in HCV+ patients.

### HCV up-regulated genes are closely related to virological and biochemical liver damage parameters, as well as to the severity of steatosis and fibrosis

Statistical analysis statistically positively correlated the HCV viremia plasma levels (VL) and antibody titer (AbT) to Adipogenin (ADIG), Adipsin (CFD), KLF15, KLF3, TCF7L2 and Twist1, either in HCV+ PBMCs or in liver tissues (**Table 4**,*p<0.05). All genes were positively correlated to liver enzymes (ALT and AST) levels, too (*p<0.05) Even more interesting was the positive correlation of all these genes with the severity of liver steatosis and Fibroscan parameters (**Table 4**).

**Table 4:** *Correlation between mRNA expression levels and virological, biochemical parameters and steatosis.

		HCV+ Liver		HCV+ PBMCs	
mRNA	IU/ml	Spearman's Rho	p	Spearman's Rho	p
Adipogenin	VL	0,2458	*0.04	0,1826	*0.046
	AbT	0,2534	*0.032	0,3430	*0.03
	AST	0,1645	*0.048	0,2598	*0.042
	ALT	0,2673	*0.023	0,2736	*0.032
	Fibro	4,5263	*0.023	0,5944	*0.031
Adipsin	VL	0,3229	*0.012	0,3909	*0.018
	AbT	0,4986	**<0.001	0,6215	**<0.001
	AST	0,2743	*0.035	0,3395	*0.026
	ALT	0,2902	*0.012	0,2472	*0.028
	Fibro	0,5868	**<0.001	0,5152	**<0.001
KLF15	VL	0,2458	*0.025	0,1826	*0.015
	AbT	0,2534	*0.04	0,3430	*0.014
	AST	0,1645	*0.021	0,2566	*0.023
	ALT	0,2673	*0.036	0,2736	*0.032
	Fibro	0,6840	**<0.001	0,5479167	**<0.001
KLF15	VL	0,31875	*0.021	0,390	*0.015
	AbT	0,3229	*0.032	0,3430	*0.011
	AST	0,2340	*0.04	0,2638	*0.014
	ALT	0,3319	*0.031	0,3368	*0.016
	Fibro	0,4569	**<0.001	0,4826	**<0.001
TCF7L2	VL	0,3805	*0.012	0,3909	*0.014
	AbT	0,3229	*0.015	0,3180	*0.023
	AST	0,2472	*0.013	0,316	*0.036
	ALT	0,4090	*0.014	0,4430	*0.021
	Fibro	0,5541	**0.001	0,594	**0.001
TWIST1	VL	0,2701	*0.018	0,2430	*0.04
	AbT	0,31666	*0.014	0,270	*0.013
	AST	0,3034	*0.016	0,3194	*0.025
	ALT	0,2604	*0.045	0,2638	*0.028
	Fibro	0,6701	**<0.001	0,5638	**<0.001

*The Spearman's rho was evaluated (84 genes and 22 biochemical data) with a Bonferroni's correction of 0.000472.

The deregulated adipogenetic genes of HBV+ patients do not display any correlation with virological parameters (data not shown).

## Discussion

Vital processes regulated by the liver including metabolism, lipid homeostasis, cellular proliferation and immune response are known to be systematically deregulated as result of persistent HCV infection [26], [27].

Our data suggest that PBMCs reflect liver adipogenesis deregulation during HCV infection. In fact, we observed a similar modulation of HCV-related lipid metabolism either in HCV+ liver tissues or lymphoid cells, highlighting the function of PBMCs as a surrogate tool of HCV steatotic liver tissues. Hepatitis C virus (HCV) is not only an hepatotropic virus, but it is also able to infect the immune system cells [28], [29], and its replication in liver are strictly related to HCV infection status in PBMCs [30]. Through the Human Adipogenesis RT^2^ Profiler PCR Array, we analyzed 84 genes involved in adipogenesis mechanism; among these 23 resulted different modulated in liver tissues and PBMCs, emphasizing the genes specificity expression in the two compartments matched. Thus, the main result was that in our HCV+ patients more than the 70% of genes, involved in the control of lipid metabolism, resulted similarly deregulated in HCV+ liver tissues and PBMCs. We confirmed the HCV-related lipid deregulation causing over-expression of: hormone sensitive Lipase (LIPE), lipoprotein lipase (LPL) and Leptin [15], as well as the modulation of genes involved in the lipid *de novo* synthesis and beta oxidation (LXR alpha, SREBP1c, FASN and ACACB) [21]. The specificity of the HCV-related adipogenic deregulated genes was supported by the fact that the adipogenic profile of HBV+ infected livers and PBMCs was deeply different from what observed in HCV+. Accordingly, the J6/JFH1 in *in vitro* HCV infection system displayed a similar adipogenesis profile, suggesting that the PBMCs modulation could really mimic what occur in the hepatocytes and was not an artifact due to lymphocyte infiltrating in the liver during HCV infection.

Some interesting molecules, involved in the control of lipid metabolism, were over-expressed. Among these, Adipogenin (ADIG) [17] that was not previously reported to be up-regulated in HCV infected patients and has an opposite behavior respect to HBV infected patients. ADIG is known to be involved not only in the adipogenesis, but also in the onset of inflammation [20].On the other end the up-regulation of the Krüppel-like transcription factors family (KLFs), in HCV infected patients, was noteworthy. KLFs are expressed in multiple tissues and regulate the expression of genes involved in glucose uptake and adipogenesis [31], [32]. KLF3 is an important regulator of several biological processes, including adipogenesis, erythropoiesis, and B cell development [33]; while, KLF15 was proposed for the regulation of the HBV gene expression and replication [34]. The role of KLF15 on HBV replication was confirmed by our experimental data. Interestingly, KLF 3 and 15 are not only implicated in adipogenesis, but also in hepatocarcinogenesis modulation [33], [35].

Finally, we appreciated the up-regulation of several genes involved in the transformation: Adipsin or complement factor D (CFD), Transcription Factor 7-like 2 (TCF7L2) and TWIST1. Adipsin is a serine protease secreted by adipocytes into the bloodstream and regulates the activation of the alternative complement pathway [36]. It could be involved in adipogenic transformation of hepatocytes [37]. Interestingly Adipsin was differently regulated in our HCV+ patients respect to HBV+ patients.

TCF7L2 has a key role in the Wnt/beta Catenin signaling [38] that is known to have an important function in the induction of HCV-related HCC and represents a critical process for the invasiveness and metastatic development of several human cancers [39]. This gene resulted positively modulated in the adipogenesis profile of HCV+ patients, while was no modulated in HBV+.

Lastly, another significant gene resulting positively modulated in the adipogenesis profile during the HCV infection is Twist1, which is able to induce the Epithelial-Mesenchymal Transition (EMT) HCV-related neoplastic transformation [40] and tumor progression [41], [42], [43].

Of noteworthy, all the molecules reported above to be deregulated, in HCV+ patients, displayed an intriguing positive correlation to virological (VL and AbT) and hepatic (ALT and AST) parameters, as well as to the severity of steatosis and fibrosis

In conclusion, the HCV+ PBMCs are able to reflect the behavior of the adipogenesis pathways of liver tissues. PBMCs might be considered a real valid support to investigate the HCV-related pathogenetic mechanisms of lipid metabolism deregulation, and to explore new therapeutical targets to overcome the HCV-related transformation.

## Supporting Information

Figure S1 **Evaluation of gene expression difference between HCV patients with different grade of fibrosis (group: F0/F1 vs group F2/F3).** Real-time analysis (mean values± S.D) of genes are expressed as fold change, and normalized with an housekeeping gene. Asterisks indicate significant difference between F0/F1 vs F2/F3 in HCV+ livers and PBMCs(*p<0,05; **p <0.001).
(TIF)

Table S1 **Microarray analysis of chronic HCV + Liver tand PBMC compared to HDs.**
(DOCX)

Table S2 **Microarray analysis of chronic HBV + Livers and PBMCs compared to HDs.**
(DOCX)

Table S3 **Differences in gene expression between HCV patients with different grade of fibrosis.** Real-time analysis (mean values± S.D) of genes are expressed as Ct and normalized with an housekeeping gene. Asterisks indicate significant difference between F0/F1 vs F2/F3 in HCV+ livers and PBMCs.
(DOC)
