# Supplementary figures and images for: Lymphocytes as Liver Damage Mirror of HCV Related Adipogenesis Deregulation
Source: PLoS One. 2014 Mar 21;9(3):e92343. doi: 10.1371/journal.pone.0092343 (PMC3962393; doi:10.1371/journal.pone.0092343)

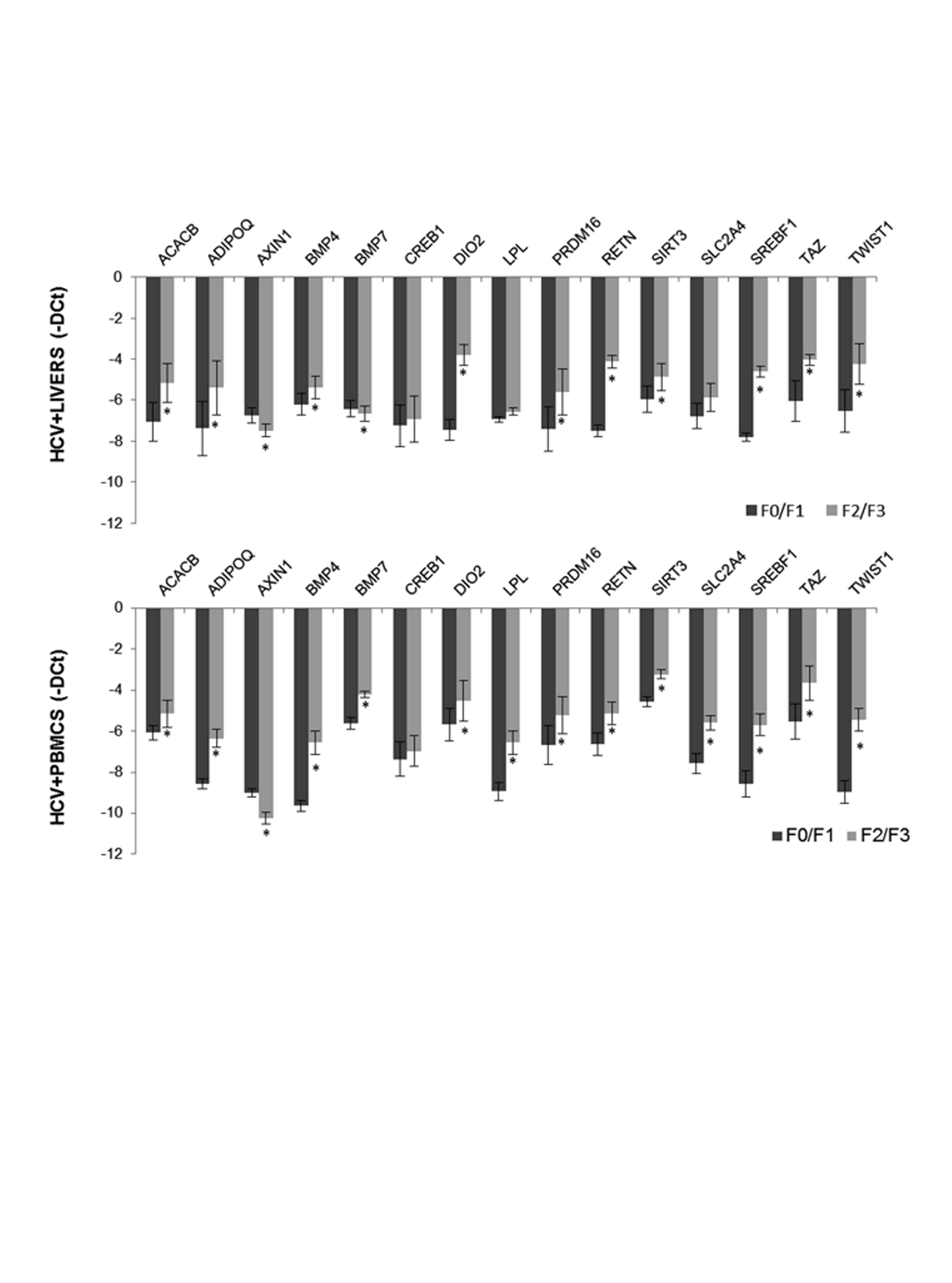

Supplement: Figure S1 — Evaluation of gene expression difference between HCV patients with different grade of fibrosis (group: F0/F1 vs group F2/F3). Real-time analysis (mean values± S.D) of genes are expressed as fold change, and normalized with an housekeeping gene. Asterisks indicate significant difference between F0/F1 vs F2/F3 in HCV+ livers and PBMCs(*p<0,05; **p <0.001). (TIF) [file pone.0092343.s001.tif]
