# Supplementary material for: Lymphocytes as Liver Damage Mirror of HCV Related Adipogenesis Deregulation
Source: PLoS One. 2014 Mar 21;9(3):e92343. doi: 10.1371/journal.pone.0092343 (PMC3962393; doi:10.1371/journal.pone.0092343)
Supplement: Table S1 — Microarray analysis of chronic HCV + Liver tand PBMC compared to HDs. (DOCX) [file pone.0092343.s002.docx]

| A) |  | | *Up-regulated genes in both HCV liver tissues and PBMCs **(47)* | | | | | | | | | | |  |
| --- | --- | --- | --- | --- | --- | --- | --- | --- | --- | --- | --- | --- | --- | --- |
|  | GENE BANK NUMBER | | | symbol | | | HCV+ liver tissues | | HCV+ PBMCs | |  | | |  |
|  |  | | |  | | | FOLD CHANGE | | FOLD CHANGE | |  | | |  |
|  |  | | P53 PATHWAYS (2) | | | | | | | | | | |  |
|  | NM_053056 | | | CCND1 | | | 4,9 | | 3,75 | | Cyclin D1 | | |  |
|  | NM_005225 | | | E2F1 | | | 7,81 | | 5,8 | | E2F transcription factor 1 | | |  |
|  |  | | WNT BETA CATENIN (7) | | | | | | | | | |  |  |
|  | NM_003394 | | | WNT10B | | | 22,96 | | 21,9 | | Wingless-type MMTV integration site family, member 10B | | |  |
|  | NM_003392 | | | WNT5A | | | 8,09 | | 5,421 | | Wingless-type MMTV integration site family, member 5A | | |  |
|  | NM_032642 | | | WNT5B | | | 4,6 | | 8,57 | | Wingless-type MMTV integration site family, member 5B | | |  |
|  | NM_030756 | | | TCF7L2 | | | 16,61 | | 20,51 | | Transcription factor 7-like 2 (T-cell specific, HMG-box) | | |  |
|  | NM_002335 | | | LRP5 | | | 3,15 | | 6,37 | | Low density lipoprotein receptor-related protein 5 | | |  |
|  | NM_012242 | | | DKK1 | | | 12,8 | | 13,42 | | Dickkopf homolog 1 (Xenopus laevis) | | |  |
|  | NM_003015 | | | SFRP5 | | | 6,69 | | 5,04 | | Secreted frizzled-related protein 5 | | |  |
|  |  | | FIBROSIS (2) | | | | | | | | | | |  |
|  | NM_004465 | | | FGF10 | | | 13,2 | | 12,44 | | Fibroblast growth factor 10 | | |  |
|  | NM_002006 | | | FGF2 | | | 8,6471 | | 6,3 | | Fibroblast growth factor 2 | | |  |
|  |  | | INSULIN PATHWAYS (4) | | | | | | | | | | |  |
|  | NM_005524 | | | HES1 | | | 4,28 | | 5,85 | | Hairy and enhancer of split 1, (NOTCH SIGNALS) | | |  |
|  | NM_000208 | | | INSR | | | 6,98 | | 7,38 | | Insulin receptor | | |  |
|  | NM_003749 | | | IRS1 | | | 3,99 | | 3,9 | | Insulin receptor substrate 2 | | |  |
|  | NM_001042 | | | GLUT4 | | | 15,28 | | 12,61 | | Glut 4 | | |  |
|  |  | | PPAR'S AND ENERGY METABOLISM (4) | | | | | | | | | | |  |
|  | NM_006238 | | | PPARD | | | 2,46 | | 2,6 | | PPARdelta | | |  |
|  | NM_013261 | | | PPARGC1A | | | 32,24 | | 26,92 | | pgc1alpha | | |  |
|  | NM_000116 | | | TAZ | | | 67,23 | | 35,54 | | Tafazzin | | |  |
|  | NM_133263 | | | PPARGC1B | | | 8,07 | | 4,49 | | Pgc1beta | | |  |
|  |  | | NUCLEAR RECEPTOR AND METABOLISM (10) | | | | | | | | | | |  |
|  | NM_001093 | | | ACACB | | | 2,96 | | 3,42 | | Acetyl-CoA carboxylase beta | | |  |
|  | NM_001442 | | | FABP4 | | | 2,57 | | 3,2 | | Fatty acid binding protein 4, adipocyte | | |  |
|  | NM_004104 | | | FASN | | | 2,93 | | 5,77 | | Fatty acid synthase | | |  |
|  | NM_005693 | | | NR1H3 | | | 5,21 | | 12,17 | | Liver X receptor alpha (LXR) | | |  |
|  | NM_004176 | | | SREBF1 | | | 8,44 | | 6,69 | | Sterol regulatory element binding transcription factor 1 | | |  |
|  | NM_021969 | | | NR0B2 | | | 4,77 | | 4,97 | | Nuclear receptor subfamily 0, group B, member 2 | | |  |
|  | NM_033131 | | | SIRT3 | | | 2,5135 | | 2,05 | | Sirtuin 3 | | |  |
|  | NM_014079 | | | KLF15 | | | 4,18 | | 5,73 | | Kruppel like factor 15 | | |  |
|  | NM_016531 | | | KLF3 | | | 3,65 | | 6,03 | | Kruppel like factor 3 | | |  |
|  | NM_000376 | | | SHH | | | 28,2218 | | 13,97 | | Sonic Hedgehog | | |  |
|  |  | | INFLAMMATION and TGF BETA PATHWAYS (11) | | | | | | | | | | |  |
|  | NM_199454 | | | PRDM16 | | | 4,65 | | 13,78 | | PR domain containing 16 | | |  |
|  | NM_130851 | | | BMP4 | | | 25,07 | | 23,47 | | Bone morphogenetic protein 4 | | |  |
|  | NM_001200 | | | BMP2 | | | 3,97 | | 6,79 | | Bone morphogenetic protein 2 | | |  |
|  | NM_130851 | | | BMP7 | | | 6,52 | | 4,51 | | Bone morphogenetic protein 7 | | |  |
|  | NM_001928 | | | CFD | | | 5,53 | | 5,75 | | Adipsin | | |  |
|  | NM_000474 | | | TWIST 1 | | | 24,07 | | 28,83 | | Twist 1 | | |  |
|  | NM_020415 | | | RETN | | | 32,51 | | 30,19 | | Resistin | | |  |
|  | NM_001018082 | | | ADIG | | | 22,78 | | 29,42 | | Adipogenin | | |  |
|  | NM_004797 | | | ADIPOQ | | | 3,12 | | 2,55 | | Adiponectin, C1Q and collagen domain containing | | |  |
|  | NM_000230 | | | LEP | | | 7,17 | | 6,38 | | Leptin | | |  |
|  | NM_000237 | | | LPL | | | 4,5 | | 9,64 | | Lipoprotein lipase | | |  |
|  |  | |  |  | | |  | |  | |  | | |  |
|  |  | | SIGNAL TRASDUCTION and REGULATION (7) | | | | | | | | | | |  |
|  | NM_000793 | | | DIO2 | | | 14,2 | | 16,82 | | Deiodinase, iodothyronine, type II | | |  |
|  | NM_003836 | | | DLK1 | | | 3,88 | | 9,27 | | Delta-like 1 homolog (Drosophila) | | |  |
|  | NM_004364 | | | CREB1 | | | 7,4597 | | 36,86 | | CAMP responsive element binding 1 | | |  |
|  | NM_005357 | | | GATA2 | | | 2,7537 | | 2,05 | | GATA 2 bindinf protein | | |  |
|  | NM_005251 | | | FOXC2 | | | 3,71 | | 2,53 | | Forkhead box C2 (MFH-1, mesenchyme forkhead 1) | | |  |
|  | NM_000399 | | | EGR2 | | | 17,45 | | 10,06 | | Early growth response 2 | | |  |
|  | NM_004364 | | | CEBPA | | | 5,3 | | 4,2 | | CCAAT/enhancer binding protein (C/EBP), alpha | | |  |
|  |  | | |  | | |  | |  | |  | | |  |
| B) |  | | *Down-regulated genes in both HCV+ liver tissues and PBMCs (6)* | | | | | | | | | | |  |
|  | GENE BANK NUMBER | | | symbol | | | HCV+ liver tissues | | HCV+ PBMCs | | | | |  |
|  |  | | |  | | | FOLD CHANGE | | FOLD CHANGE | | | | |  |
|  |  | | |  | | |  | |  | |  | | |  |
|  | NM_004089 | | | TSC22D3 | | | 0 | | 0 | | TSC22 domain family, member 3 | | |  |
|  | NM_033131 | | | WNT3A | | | 0,0003 | | 0,0001 | | Wingless-type MMTV integration site family, member 3A | | |  |
|  | NM_021833 | | | UCP1 | | | 0,001 | | 0,0016 | | Uncoupling protein 1 (mitochondrial, proton carrier) | | |  |
|  | NM_000376 | | | VDR | | | 0 | | 0 | | Vitamin D (1,25- dihydroxyvitamin D3) receptor | | |  |
|  | NM_005430 | | | WNT1 | | | 0 | | 0 | | Wingless-type MMTV integration site family, member 1 | | |  |
|  | NM_003502 | | | AXIN1 | | | 0,3908 | | 0,3196 | | axin1 | | |  |
| C) | | *No-modulated genes in both HCV+ liver tissues and PBMCs (8)* | | | | | | | | | |  | | |
|  | | GENE BANK NUMBER | | | symbol |  | | HCV+ liver tissues | | HCV+ PBMCs | |  | | |
|  | |  | | |  |  | | FOLD CHANGE | | FOLD CHANGE | |  | | |
|  | | NM_005572 | | |  | LMNA | | 1,0935 | | 0,9968 | | Lamin A/C | | |
|  | | NM_004235 | | |  | KLF4 | | 0,9119 | | 0,7112 | | Kruppel-like factor 4 (gut) | | |
|  | | NM_000389 | | |  | CDKN1A | | 0,9962 | | 0,9064 | | Cyclin-dependent kinase inhibitor 1A (p21, Cip1) | | |
|  | | NM_005011 | | |  | NRF1 | | 0,8016 | | 0,6815 | | Nuclear respiratory factor 1 | | |
|  | | NM_012238 | | |  | SIRT1 | | 1,4083 | | 1,458 | | Sirtuin 1 | | |
|  | | NM_012237 | | |  | SIRT2 | | 0,6538 | | 0,9242 | | Sirtuin 2 | | |
|  | | NM_005417 | | |  | SRC | | 1, 284 | | 0,8933 | | V-src sarcoma viral oncogene | | |
|  | | NM_001315 | | |  | MAPK14 | | 0,9025 | | 0,8518 | | Mitogen-activated protein kinase 14 | | |
| D) | | *Analysis of modulated genes in only HCV liver tissues or PBMCs (23)* | | | | | | | | | |  | | |
|  | | GENE BANK NUMBER | | | Symbol |  | | HCV+ liver tissues | | HCV+ PBMCs | |  | | |
|  | |  | | |  |  | | FOLD CHANGE | | FOLD CHANGE | |  | | |
|  | | NM_000024 | | |  | ADRB2 | | 0,00 | | nm | | Adrenergic, beta-2-, receptor, surface | | |
|  | | NM_000029 | | |  | AGT | | 159,07 | | nm | | Angiotensinogen (serpin peptidase inhibitor, clade A | | |
|  | | NM_001147 | | |  | ANGPT2 | | Nm | | 2,76 | | Angiopoietin 2 | | |
|  | | NM_000075 | | |  | CDK4 | | 4,50 | | nm | | Cyclin-dependent kinase 4 | | |
|  | | NM_004064 | | |  | CDKN1B | | 0,39 | | nm | | Cyclin-dependent kinase inhibitor 1B (p27, Kip1) | | |
|  | | NM_005194 | | |  | CEBPB | | 2,03 | | nm | | CCAAT/enhancer binding protein (C/EBP), beta | | |
|  | | NM_005195 | | |  | CEBPD | | Nm | | 3,39 | | CCAAT/enhancer binding protein (C/EBP), delta | | |
|  | | NM_004083 | | |  | DDIT3 | | 2,13 | | 0,44 | | DNA-damage-inducible transcript 3 | | |
|  | | NM_000800 | | |  | FGF1 | | 2,72 | | nm | | Fibroblast growth factor 1 (acidic) | | |
|  | | NM_002015 | | |  | FOXO1 | | Nm | | 2,26 | | Forkhead box O1 | | |
|  | | NM_002051 | | |  | GATA3 | | 0,41 | | 2,24 | | GATA binding protein 3 | | |
|  | | NM_003749 | | |  | IRS2 | | Nm | | 4 | | Insulin receptor substrate 2 | | |
|  | | NM_002228 | | |  | JUN | | Nm | | 4,88 | | Jun proto-oncogene | | |
|  | | NM_016270 | | |  | KLF2 | | 0,05 | | nm | | Kruppel-like factor 2 (lung) | | |
|  | | NM_005357 | | |  | LIPE | | Nm | | 6,04 | | Lipase, hormone-sensitive | | |
|  | | NM_005036 | | |  | PPARA | | 16,49 | | nm | | Peroxisome proliferator-activated receptor alpha | | |
|  | | NM_015869 | | |  | PPARG | | 6,03 | | nm | | Peroxisome proliferator-activated receptor gamma | | |
|  | | HS_446678 | | |  | NCOA2 | | 4,07 | | nm | | Nuclear receptor coactivator 2 | | |
|  | | HS_137510 | | |  | NCOR2 | | 2,59 | | nm | | Nuclear receptor corepressor 2 | | |
|  | | NM_000321 | | |  | RB1 | | 16,52 | | nm | | Retinoblastoma 1 | | |
|  | | NM_175636 | | |  | RUNX1T1 | | 2,75 | | nm | | Runt-related transcription factor 1 (cyclin D-related) | | |
|  | | NM_002957 | | |  | RXRA | | 30,05 | | nm | | Retinoid X receptor, alpha | | |
|  | | NM_003012 | | |  | SFRP1 | | 8,82 | | nm | | Secreted frizzled-related protein 1 | | |

FOOTNOTES The results are the fold change respect to HDs; ** the up-regulated genes were divided in respect their functions
