# Supplementary material for: Lymphocytes as Liver Damage Mirror of HCV Related Adipogenesis Deregulation
Source: PLoS One. 2014 Mar 21;9(3):e92343. doi: 10.1371/journal.pone.0092343 (PMC3962393; doi:10.1371/journal.pone.0092343)
Supplement: Table S2 — Microarray analysis of chronic HBV + Livers and PBMCs compared to HDs. (DOCX) [file pone.0092343.s003.docx]

Table 3: Adipogenesis modulation in chronic HBV + Livers and PBMCs compared to HDs

| \| **GeneBank** \|  \| HBV + LIVERS \| HBV PBMCS \|  \| \| --- \| --- \| --- \| --- \| --- \| \| NM_001093 \| ACACB \| 0,00295 \| 0,0017 \| Acetyl-CoA carboxylase beta \| \| NM_001018082 \| ADIG \| 0,00955 \| 0,0083 \| Adipogenin \| \| NM_004797 \| ADIPOQ \| 0,00235 \| 0,0011 \| Adiponectin, C1Q and collagen domain containing \| \| NM_000024 \| ADRB2 \| 0,00135 \| 0,0001 \| Adrenergic, beta-2-, receptor, surface \| \| NM_000029 \| AGT \| 0,00195 \| 0,0007 \| Angiotensinogen (serpin peptidase inhibitor, clade A, member 8) \| \| NM_001147 \| ANGPT2 \| 0,00185 \| 0,0006 \| Angiopoietin 2 \| \| NM_003502 \| AXIN1 \| 0,00145 \| 0,0002 \| Axin 1 \| \| NM_001200 \| BMP2 \| 0,00925 \| 0,008 \| Bone morphogenetic protein 2 \| \| NM_130851 \| BMP4 \| 0,01495 \| 0,025 \| Bone morphogenetic protein 4 \| \| NM_001719 \| BMP7 \| 0,00645 \| 0,056 \| Bone morphogenetic protein 7 \| \| NM_053056 \| CCND1 \| 0,00555 \| 0,0043 \| Cyclin D1 \| \| NM_000075 \| CDK4 \| 0,00195 \| 0,0007 \| Cyclin-dependent kinase 4 \| \| NM_000389 \| CDKN1A \| 0,00135 \| 0,0001 \| Cyclin-dependent kinase inhibitor 1A (p21, Cip1) \| \| NM_004064 \| CDKN1B \| 0,00125 \| 0 \| Cyclin-dependent kinase inhibitor 1B (p27, Kip1) \| \| NM_004364 \| CEBPA \| 0,00235 \| 0,0137 \| CCAAT/enhancer binding protein (C/EBP), alpha \| \| NM_005194 \| CEBPB \| nt \| 0,0089 \| CCAAT/enhancer binding protein (C/EBP), beta \| \| NM_005195 \| CEBPD \| nt \| 0,0016 \| CCAAT/enhancer binding protein (C/EBP), delta \| \| NM_001928 \| CFD \| 0,00535 \| 0,0003 \| Complement factor D (adipsin) \| \| NM_004379 \| CREB1 \| 0,00195 \| 0,0014 \| CAMP responsive element binding protein 1 \| \| NM_004083 \| DDIT3 \| 0,00155 \| 0,0089 \| DNA-damage-inducible transcript 3 \| \| NM_000793 \| DIO2 \| 0,00875 \| 0,0016 \| Deiodinase, iodothyronine, type II \| \| NM_012242 \| DKK1 \| 0,00655 \| 0,0089 \| Dickkopf homolog 1 (Xenopus laevis) \| \| NM_003836 \| DLK1 \| 0,00575 \| 0,0016 \| Delta-like 1 homolog (Drosophila) \| \| NM_005225 \| E2F1 \| 0,01495 \| 0,0003 \| E2F transcription factor 1 \| \| NM_000399 \| EGR2 \| 0,01015 \| 0,0075 \| Early growth response 2 \| \| NM_001442 \| FABP4 \| 0,03065 \| 0,0016 \| Fatty acid binding protein 4, adipocyte \| \| NM_004104 \| FASN \| 0,02935 \| 0,0003 \| Fatty acid synthase \| \| NM_000800 \| FGF1 \| 0,03045 \| 0,0014 \| Fibroblast growth factor 1 (acidic) \| \| NM_004465 \| FGF10 \| 0,03735 \| 0,0083 \| Fibroblast growth factor 10 \| \| NM_002006 \| FGF2 \| 0,03055 \| 0,0015 \| Fibroblast growth factor 2 (basic) \| \| NM_005251 \| FOXC2 \| 0,03165 \| 0,0026 \| Forkhead box C2 (MFH-1, mesenchyme forkhead 1) \| \| NM_002015 \| FOXO1 \| 0,02925 \| 0,0002 \| Forkhead box O1 \| \| NM_032638 \| GATA2 \| 0,03065 \| 0,0016 \| GATA binding protein 2 \| \| NM_002051 \| GATA3 \| 0,02935 \| 0,0011 \| GATA binding protein 3 \| \| NM_005524 \| HES1 \| 0,37495 \| 0,0001 \| Hairy and enhancer of split 1, (Drosophila) \| \| NM_000208 \| INSR \| 0,15025 \| 0,0003 \| Insulin receptor \| \| NM_005544 \| IRS1 \| 0,0016 \| 0,03065 \| Insulin receptor substrate 1 \| \| NM_003749 \| IRS2 \| 0,0003 \| 0,02935 \| Insulin receptor substrate 2 \| \| NM_002228 \| JUN \| 0,0942 \| 0,0003 \| Jun proto-oncogene \| \| NM_014079 \| KLF15 \| 3,56 \| 2,54 \| Kruppel-like factor 15 \| \| NM_016270 \| KLF2 \| 0,0942 \| 0,0053 \| Kruppel-like factor 2 (lung) \| \| NM_016531 \| KLF3 \| 0,0949 \| 0,001 \| Kruppel-like factor 3 (basic) \| \| NM_004235 \| KLF4 \| 5,65 \| 4,74 \| Kruppel-like factor 4 (gut) \| \| NM_000230 \| LEP \| 0,1143698 \| 0,0468 \| Leptin \| \| NM_005357 \| LIPE \| 0,0815698 \| 0,014 \| Lipase, hormone-sensitive \| \| NM_005572 \| LMNA \| 1,56 \| 1,11 \| Lamin A/C \| \| NM_000237 \| LPL \| 0,0322698 \| 0,0067 \| Lipoprotein lipase \| \| NM_002335 \| LRP5 \| 6,33 \| 5,45 \| Low density lipoprotein receptor-related protein 5 \| \| NM_001315 \| MAPK14 \| 2 \| 3,2 \| Mitogen-activated protein kinase 14 \| \| NM_006540 \| NCOA2 \| 4,56 \| 3,65 \| Nuclear receptor coactivator 2 \| \| NM_006312 \| NCOR2 \| 0,0265698 \| 0,001 \| Nuclear receptor corepressor 2 \| \| NM_021969 \| NR0B2 \| 0,0344698 \| 0,0089 \| Nuclear receptor subfamily 0, group B, member 2 \| \| NM_005693 \| NR1H3 \| 0,0635698 \| 0,038 \| Nuclear receptor subfamily 1, group H, member 3 \| \| NM_005011 \| NRF1 \| 0,0506698 \| 0,0251 \| Nuclear respiratory factor 1 \| \| NM_005036 \| PPARA \| 2,56 \| 3,45 \| Peroxisome proliferator-activated receptor alpha \| \| NM_006238 \| PPARD \| 5,45 \| 5,98 \| Peroxisome proliferator-activated receptor delta \| \| NM_015869 \| PPARG \| 0,0304698 \| 0,0049 \| Peroxisome proliferator-activated receptor gamma \| \| NM_013261 \| PPARGC1A \| 0,6574698 \| 0,6319 \| Peroxisome proliferator-activated receptor gamma, coactivator 1 alpha \| \| NM_133263 \| PPARGC1B \|  \|  \| Peroxisome proliferator-activated receptor gamma, coactivator 1 beta \| \| NM_199454 \| PRDM16 \| 0,0437698 \| 0,0182 \| PR domain containing 16 \| \| NM_000321 \| RB1 \| 6,15 \| 6,45 \| Retinoblastoma 1 \| \| NM_020415 \| RETN \| 21 \| 19,88 \| Resistin \| \| NM_175636 \| RUNX1T1 \| 0,0374698 \| 0,0119 \| Runt-related transcription factor 1; translocated to, 1 (cyclin D-related) \| \| NM_002957 \| RXRA \| 0,3220698 \| 0,2965 \| Retinoid X receptor, alpha \| \| NM_003012 \| SFRP1 \| 0,0494698 \| 0,0239 \| Secreted frizzled-related protein 1 \| \| NM_003015 \| SFRP5 \| 6,88 \| 5,09 \| Secreted frizzled-related protein 5 \| \| NM_000193 \| SHH \| 0,2432698 \| 0,2177 \| Sonic hedgehog \| \| NM_012238 \| SIRT1 \| 2,95 \| 1,87 \| Sirtuin 1 \| \| NM_012237 \| SIRT2 \| 3,65 \| 4,45 \| Sirtuin 2 \| \| NM_012239 \| SIRT3 \| 3,56 \| 3 \| Sirtuin 3 \| \| NM_001042 \| SLC2A4 \| 0,066 \| 0,0405 \| Solute carrier family 2 (facilitated glucose transporter), member 4 \| \| NM_005417 \| SRC \| nt \| 5,00 \| V-src sarcoma (Schmidt-Ruppin A-2) viral oncogene homolog (avian) \| \| NM_004176 \| SREBF1 \| 12,36 \| 11,02 \| Sterol regulatory element binding transcription factor 1 \| \| NM_000116 \| TAZ \| 1,45 \| 3,00 \| Tafazzin \| \| NM_030756 \| TCF7L2 \| 1,54 \| 0,73 \| Transcription factor 7-like 2 (T-cell specific, HMG-box) \| \| NM_004089 \| TSC22D3 \| 0,0255698 \| 0 \| TSC22 domain family, member 3 \| \| NM_000474 \| TWIST1 \| 0,0392698 \| 0,0137 \| Twist homolog 1 (Drosophila) \| \| NM_021833 \| UCP1 \| 0,0256698 \| 0,0001 \| Uncoupling protein 1 (mitochondrial, proton carrier) \| \| NM_000376 \| VDR \| 0,0255698 \| 0 \| Vitamin D (1,25- dihydroxyvitamin D3) receptor \| \| NM_005430 \| WNT1 \| 0,0255698 \| 0 \| Wingless-type MMTV integration site family, member 1 \| \| NM_003394 \| WNT10B \| 5,56 \| 6,64 \| Wingless-type MMTV integration site family, member 10B \| \| NM_033131 \| WNT3A \| nt \| 0 \| Wingless-type MMTV integration site family, member 3A \| \| NM_003392 \| WNT5A \| 0,6597698 \| 0,6342 \| Wingless-type MMTV integration site family, member 5A \| \| NM_032642 \| WNT5B \| 0,2764698 \| 0,2509 \| Wingless-type MMTV integration site family, member 5B \| |  |  |  |  |
| --- | --- | --- | --- | --- | --- | --- | --- | --- | --- | --- | --- | --- | --- | --- | --- | --- | --- | --- | --- | --- | --- | --- | --- | --- | --- | --- | --- | --- | --- | --- | --- | --- | --- | --- | --- | --- | --- | --- | --- | --- | --- | --- | --- | --- | --- | --- | --- | --- | --- | --- | --- | --- | --- | --- | --- | --- | --- | --- | --- | --- | --- | --- | --- | --- | --- | --- | --- | --- | --- | --- | --- | --- | --- | --- | --- | --- | --- | --- | --- | --- | --- | --- | --- | --- | --- | --- | --- | --- | --- | --- | --- | --- | --- | --- | --- | --- | --- | --- | --- | --- | --- | --- | --- | --- | --- | --- | --- | --- | --- | --- | --- | --- | --- | --- | --- | --- | --- | --- | --- | --- | --- | --- | --- | --- | --- | --- | --- | --- | --- | --- | --- | --- | --- | --- | --- | --- | --- | --- | --- | --- | --- | --- | --- | --- | --- | --- | --- | --- | --- | --- | --- | --- | --- | --- | --- | --- | --- | --- | --- | --- | --- | --- | --- | --- | --- | --- | --- | --- | --- | --- | --- | --- | --- | --- | --- | --- | --- | --- | --- | --- | --- | --- | --- | --- | --- | --- | --- | --- | --- | --- | --- | --- | --- | --- | --- | --- | --- | --- | --- | --- | --- | --- | --- | --- | --- | --- | --- | --- | --- | --- | --- | --- | --- | --- | --- | --- | --- | --- | --- | --- | --- | --- | --- | --- | --- | --- | --- | --- | --- | --- | --- | --- | --- | --- | --- | --- | --- | --- | --- | --- | --- | --- | --- | --- | --- | --- | --- | --- | --- | --- | --- | --- | --- | --- | --- | --- | --- | --- | --- | --- | --- | --- | --- | --- | --- | --- | --- | --- | --- | --- | --- | --- | --- | --- | --- | --- | --- | --- | --- | --- | --- | --- | --- | --- | --- | --- | --- | --- | --- | --- | --- | --- | --- | --- | --- | --- | --- | --- | --- | --- | --- | --- | --- | --- | --- | --- | --- | --- | --- | --- | --- | --- | --- | --- | --- | --- | --- | --- | --- | --- | --- | --- | --- | --- | --- | --- | --- | --- | --- | --- | --- | --- | --- | --- | --- | --- | --- | --- | --- | --- | --- | --- | --- | --- | --- | --- | --- | --- | --- | --- | --- | --- | --- | --- | --- | --- | --- | --- | --- | --- | --- | --- | --- | --- | --- | --- | --- | --- | --- | --- | --- | --- | --- | --- | --- | --- | --- | --- | --- | --- | --- | --- | --- | --- | --- | --- | --- | --- | --- | --- | --- | --- | --- | --- | --- | --- | --- | --- | --- | --- | --- | --- | --- | --- | --- | --- | --- | --- | --- | --- | --- | --- | --- | --- | --- | --- | --- | --- | --- | --- | --- | --- | --- | --- | --- | --- | --- | --- | --- |
|  |  |  |  |  |
|  |  |  |  |  |
|  |  |  |  |  |
|  |  |  |  |  |
|  |  |  |  |  |
|  |  |  |  |  |
|  |  |  |  |  |
|  |  |  |  |  |
