# Supplementary material for: Lymphocytes as Liver Damage Mirror of HCV Related Adipogenesis Deregulation
Source: PLoS One. 2014 Mar 21;9(3):e92343. doi: 10.1371/journal.pone.0092343 (PMC3962393; doi:10.1371/journal.pone.0092343)
Supplement: Table S3 — Differences in gene expression between HCV patients with different grade of fibrosis. Real-time analysis (mean values± S.D) of genes are expressed as Ct and normalized with an housekeeping gene. Asterisks indicate significant difference between F0/F1 vs F2/F3 in HCV+ livers and PBMCs. (DOC) [file pone.0092343.s004.doc]

|  | | | | | | | | | | |
| --- | --- | --- | --- | --- | --- | --- | --- | --- | --- | --- |
|  | HCV LIVERS | | | | | HCV PBMCs | | | | |
| Fibroscan | F0/F1 (25) | | F2/F3 (15) | | P* | F0/F1 (25) | | F2/F3 (15) | | P* |
| -(Ct-BetaACT) | average | ±ds | average | ±ds |  | Average | ±ds | average | ±ds |  |
| ACACB | -7,07 | 0,95 | -5,15 | 0,95 | 0,00 | -6,07 | 0,36 | -5,15 | 0,65 | 0.001 |
| ADIPOQ | -7,37 | 1,33 | -5,40 | 1,33 | 0,019 | -8,56 | 0,25 | -6,35 | 0,45 | 0.05 |
| AXIN1 | -6,75 | 0,36 | -7,48 | 0,31 | 0,047 | -9,00 | 0,21 | -10,23 | 0,29 | 0.038 |
| BMP4 | -6,22 | 0,53 | -5,39 | 0,56 | 0,021 | -9,63 | 0,26 | -6,56 | 0,58 | 0.05 |
| BMP7 | -6,44 | 0,39 | -6,68 | 0,38 | 0,036 | -5,62 | 0,28 | -4,21 | 0,15 | 0.025 |
| CREB1 | -7,24 | 1,02 | -6,92 | 1,12 | 0,021 | -7,36 | 0,85 | -6,96 | 0,74 | 0.031 |
| DIO2 | -7,44 | 0,50 | -3,80 | 0,50 | 0,037 | -5,68 | 0,78 | -4,52 | 1,00 | 0.035 |
| LPL | -6,95 | 0,15 | -6,56 | 0,18 | 0,024 | -8,94 | 0,45 | -6,56 | 0,56 | 0.04 |
| PRDM16 | -7,41 | 1,09 | -5,61 | 1,12 | 0,023 | -6,68 | 0,96 | -5,22 | 0,89 | 0.036 |
| RETN | -7,50 | 0,29 | -4,12 | 0,32 | 0,001 | -6,63 | 0,54 | -5,12 | 0,54 | 0.001 |
| SIRT3 | -5,96 | 0,64 | -4,88 | 0,65 | 0,00 | -4,56 | 0,25 | -3,22 | 0,21 | 0.001 |
| SLC2A4 | -6,78 | 0,62 | -5,88 | 0,69 | 0,00 | -7,56 | 0,48 | -5,59 | 0,36 | 0.025 |
| SREBF1 | -7,82 | 0,21 | -4,61 | 0,25 | 0,00 | -8,56 | 0,63 | -5,69 | 0,54 | 0.031 |
| TAZ | -6,03 | 0,99 | -4,04 | 0,25 | 0,010 | -5,54 | 0,85 | -3,65 | 0,85 | 0.005 |
| TWIST1 | -6,54 | 1,04 | -4,23 | 0,99 | 0,041 | -8,96 | 0,56 | -5,44 | 0,56 | 0.005 |

**Table S3**: Differences in gene expression between HCV patients with different grade of fibrosis
